# Does a Lack of Early Intensive Socialisation with Humans Exclude Goats from Participating in Animal-Assisted Services?

**DOI:** 10.3390/ani16040564

**Published:** 2026-02-11

**Authors:** Wiktoria Janicka, Kamila Janicka, Patrycja Magdalena Masier, Agnieszka Ziemiańska, Iwona Rozempolska-Rucińska

**Affiliations:** Institute of Biological Basis of Animal Production, University of Life Sciences in Lublin, 20-950 Lublin, Poland; wiktoria.janicka@up.edu.pl (W.J.); patrycja.masier@up.edu.pl (P.M.M.); agnieszka.ziemianska@up.edu.pl (A.Z.); iwona.rucinska@up.edu.pl (I.R.-R.)

**Keywords:** animal-assisted services, goat-assisted services, human–animal interactions, socialisation, sociability, social support

## Abstract

Socialisation with humans and willingness to interact with them are essential traits for animals involved in animal-assisted services (AAS). This study examined whether goats without prior intensive socialisation with humans may show predispositions to AAS in terms of sociability towards people. Ten goats underwent four tests: (1) acceptance of human approach and touch, voluntary approach to (2) a passive and (3) an active human, and (4) a novel sound fear test. Tests 1–3 were conducted with the caretaker and a familiar neutral person, and 4 with the neutral person. The goats generally accepted being approached and touched but showed little interest in interacting voluntarily with humans. Goats’ responses did not differ between the caretaker and the neutral person (*p* > 0.05). In the fear test, goats escaped less often and returned more quickly after the sound playback when a human was present (*p* < 0.01). Female goats exhibited higher sociability towards humans. Overall, the goats demonstrated some predispositions to AAS, such as tolerance of human contact and a tendency to perceive humans as a source of support during stress. However, these traits alone are insufficient for participation in AAS. Additional training is needed to increase the goats’ willingness to initiate interactions with people.

## Simple Summary

Animal-assisted services (AAS) refer to any intervention that incorporates animals into a therapeutic or supportive process or environment. The animal’s motivation and enjoyment in interacting with humans make the experience particularly pleasant for all parties involved and have important consequences for animal welfare. This study explored whether adult goats without prior frequent contact with humans may exhibit social predispositions toward inclusion in AAS. The goats generally accepted being approached and touched by both their caretaker and another familiar person who visited them less often. They were also calmer during a stressful event; however, they rarely initiated interactions with humans. Female goats voluntarily approached a passive human and accepted being approached and touched with a higher probability than male goats. Generally, the goats showed some predisposition to participate in AAS, but further training is needed. Such training should focus on improving the goats’ acceptance of human presence and their motivation to voluntarily engage in interactions.:

## 1. Introduction

Animal-assisted services (AAS) comprise practices conducted under the guidance of specialists that intentionally incorporate specially qualified animals to support therapeutic, educational, or supportive goals for humans [1,2]. Historically, AAS focused primarily on dogs and horses due to their long history of domestication, extensive training suitability, and substantial evidence supporting their use in therapeutic settings. More recently, however, research and practice have expanded to include a wider range of domesticated species, including goats, sheep, donkeys, alpacas, llamas, and cattle [2]. Although AAS popularity continues to increase, studies evaluating the animal component, especially farm animals, are still limited. Much of the existing evidence comes from companion animals, and the transferability of this knowledge is uncertain [3,4]. Farm-animal-based AAS are emerging, particularly in care-farm contexts, where clients engage with animals through caregiving activities such as feeding, grooming, and routine husbandry [4,5,6]. These settings may offer unique benefits, including multisensory engagement, structured routines, opportunities for physical activity, and stimulation of social behaviour [4]. A precondition for incorporating any species into AAS is assessing whether individuals exhibit appropriate temperament and welfare suitability [7,8]. Each species has its own expectations for interacting with humans, and even naturally shy or reclusive animals can still flourish during such encounters. However, any contact must be guided by the animal’s individual needs and comfort level and should only occur when the animal is willing to engage [6]. Welfare considerations are critical, as repeated non-voluntary contact with unfamiliar humans may induce stress, particularly if animals are not temperamentally suited or inadequately socialised [9,10].

One of the key traits influencing an animal’s suitability for AAS is its sociability toward humans [11], reflected in behaviours documented across studies, such as voluntarily approaching people, remaining in proximity, accepting physical contact, and displaying affiliative or interaction-seeking responses during human–animal encounters [12,13,14,15]. In line with IAHAIO recommendations, animals involved in AAS should demonstrate a willingness to engage positively with humans and accept tactile interaction, such as stroking or brushing, which can enhance the quality of human–animal interaction [11]. This aspect is often considered within the broader domain of human–animal interaction (HAI), which encompasses the reciprocal behavioural, emotional, and physiological exchanges occurring between humans and animals [16]. Notably, voluntary participation and the ability to balance proximity and distance have important welfare implications: animals that choose contact and can withdraw when needed are less likely to experience stress. Therefore, respecting individuals’ boundaries is crucial to safeguarding their well-being. A key factor determining whether an animal can successfully participate in AAS is its history of socialisation with humans. Recent research shows that goats with frequent, positive human contact tend to be calmer, more approachable, and more willing to initiate proximity than those with limited interaction [15,17]. Limited socialisation may reduce human-directed behaviour and stress-buffering effects, as goats with fewer prior interactions show less affiliative behaviour and weaker responses to human social support [18,19]. Although early-life experiences clearly contribute to sociability, older goats can also improve their responses to humans through consistent, positive handling; however, the extent of this improvement is highly variable and depends on temperament and previous experiences, as demonstrated in long-term developmental studies [20]. Furthermore, validated behavioural tools, such as the Familiar Human Approach Test, highlight the importance of considering past handling when evaluating human–animal relationships, especially in pasture-based systems, where animals generally maintain greater distance from humans [21].

To evaluate sociability and HAI in livestock, researchers often use structured behavioural tests that identify individual differences in fearfulness, boldness, curiosity, and overall temperament. Temperament is especially relevant to AAS because animals vary greatly in their ability to cope with novelty, handling, and unpredictable human behaviour [22,23]. Individuals who respond calmly and consistently across contexts are more likely to interact safely and voluntarily with unfamiliar people. Standardised evaluations—including variations in the Human Approach Test (HAT) and measures of latency to first contact—are widely used to assess voluntary approach, avoidance distance, and tolerance of tactile interaction. These metrics have been incorporated into ruminant welfare protocols such as the AWIN protocol for goats [24], in which HAI indicators play a central role in evaluating sociability and ease of handling. In AWIN, latency-based measures and a voluntary approach toward a familiar human provide information about fear reactivity and affiliative motivation. These methods are considered ecologically meaningful because a willingness to seek proximity reflects intrinsic motivation rather than forced compliance. However, most validated HAI measures were developed for intensive or semi-intensive housing, and, therefore, their limited applicability under pasture-based conditions should be taken into account. For example, it was observed that goats on pasture generally maintain greater distances from humans, reducing test sensitivity [21].

Goats are increasingly recognised as promising candidates for AAS. They are described as intelligent, affectionate, curious, and often highly motivated to interact with humans [25,26]. Individual goats may display spontaneous proximity-seeking, voluntary following, and apparent enjoyment of tactile contact, such as stroking and grooming; some reports describe licking or nibbling familiar people as a form of affiliative exploration [15,26]. Their relatively small size and calm demeanour enable safe interaction with diverse populations, including wheelchair users [17,25]. Despite their suitability, scientific understanding of human–goat sociability and communication remains limited. Evidence suggests that goats use tactile and gustatory signals in social interaction, though their functional significance is not yet well understood [26]. Social behaviour is a fundamental component of goat ethology, facilitating group cohesion, access to resources, and predator avoidance [27]. Consequently, sociability toward humans should ideally be evaluated in the group context typical of natural goat social environments rather than under artificial constraints. However, despite increasing interest in goats as potential partners in AAS, no standardised criteria exist for selecting suitable individuals. To the authors’ knowledge, no previous studies have evaluated human-directed sociability in goats under pasture conditions for AAS applications.

The study was conducted as part of a planned training programme to prepare the goats for classes with students and for participation in goat-assisted activities. It provides a preliminary assessment of goats’ sociability toward humans, specifically approach acceptance, proximity seeking, tolerance of touch, and seeking social support from humans, in a pasture-based setting, emphasising a natural group context and voluntary participation. Using simple human–animal interaction tests, the authors aimed to assess (1) if the goats without any previous training and intensive socialisation with humans (e.g., petting or playing) will show predispositions to AAS in the context of sociability towards humans and (2) whether, at the initial stage of the training, the test outcomes may depend on the person conducting them. Interactions with unfamiliar humans are an unavoidable component of AAS, and therefore, animals’ responses to unknown humans are highly relevant for evaluating their welfare in the context of AAS. However, since encounters with humans may constitute a source of stress for animals depending on previous experiences or low levels of socialisation with humans [9,10,28,29], we decided to compare responses to a caretaker (daily contact, possible association with feeding) and a neutral person (familiar, but less frequent contact, no feeding context) at this early stage of training. The discussion also addresses further possible training approaches for the studied group of goats aimed at AAS.

## 2. Materials and Methods

### 2.1. Animals and Housing

The study included 10 goats of different ages, sexes, and breeds. There were five males and five females of various breeds, seven of whom were adults (2–10 years old) and three were younger (6–8 months old). All the goats were kept together for a month on pasture at the University of Life Sciences in Lublin, Poland (Lublin Voivodeship), known as the Animal Zone. They were supposed to be used during classes with students on animal care, welfare, and training, and, in the future, possibly also in animal-assisted services (AAS) with visitors of different ages. None of the goats had previously been used in AAS, undergone training in this field, or been tested for predisposition to AAS. They arrived at the Animal Zone, accustomed to the presence of a human during standard procedures, such as feeding, bedding, farm clean-up (no restraint; the goats were allowed to move freely), and veterinary care (short-term restraint), where they had previously been kept. Only two of them were in the past led out on a lead attached to a halter to the paddock and occasionally brushed. However, no individual had ever been subjected to intensive socialisation with humans, including petting, training, or playing. After a prior adaptation period, such activities were to be undertaken in the Animal Zone.

**Figure 1:**
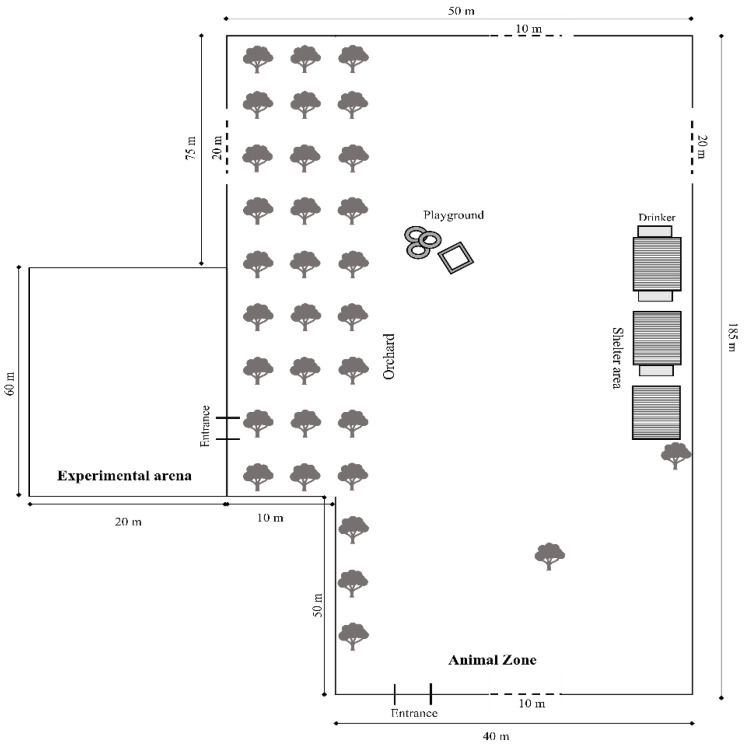
Scheme of the pasture where the goats are kept. Specifically, the Animal Zone with its elements (orchard, playground, shelter area, and drinkers) and the Experimental Zone where the tests were conducted are shown. The dashed lines indicate sections of the diagram that have been abbreviated for visual purposes.

The Animal Zone provided the animals protection from adverse weather and access to environmental enrichment (Figure 1). It covered 0.92 hectares, and a smaller pasture (0.12 hectares) was also attached to the western side of the zone and periodically made available to the animals. During the study period, it served as an experimental arena and was open to goats for at least two hours daily beyond the testing hours, so they would treat it as part of the zone and behave naturally.

On the eastern edge of the zone, there were three wooden shelters (20 m^2^ each) with four walls and a 2 m wide entrance on the front wall. The shelters were bedded with wheat straw. There were three watering sites directly next to the shelters. On the western side of the zone, there was a strip of fruit trees approximately 10 m wide, stretching along the entire length of the zone. In the central area of the zone, a playground was built, consisting of a small house and boards attached to tyres for climbing. During the winter, the goats were to be moved to a goat shed (60 m^2^) with access to an outdoor area (100 m^2^).

Animals were handled by two caretakers: a man and a woman. They were responsible for the daily care of the animals, their general health, preparing concentrated feed, delivering hay, feeding the animals, providing fresh water and bedding, and cleaning work within the Animal Zone. The man was the main carer and looked after the goats every day. The woman helped with care 3–4 times a week. The goats were fed meadow hay, a mixture of root vegetables (carrots and beetroots), dried herbs, and concentrated feed for small ruminants once daily and had access to grass within the zone. Hay was provided in sufficient quantities for one day. All animals participating in the study were healthy, and none of the goats showed any signs of somatic disease or behavioural disorders.

### 2.2. Study Design

#### 2.2.1. Adaptation Period

The study consisted of two stages: (1) an adaptation period and (2) behavioural tests. The focus was on human–animal interaction tests, as willingness to interact with humans is the main goal during AAS [2,17]. At the beginning, two simple tests were conducted: a voluntary approach to a human and acceptance of a human approach. The approach tests were performed with the participation of the feeding person (FP; male, main caretaker of the animals) and a neutral person (NP; female, non-feeding, academic staff, one of the visitors to the Animal Zone, usually three times a week). The purpose of this procedure was to determine whether the goats would associate a given person with food and, if so, whether this association could influence the results of the AAS predisposition tests. While testing, FP and NP wore clothing in similar, muted colours. FP continued performing his routine duties throughout the study, whereas NP continued to visit the zone 3–4 times per week. Subsequently, after analysing the results of the approach tests, the fear test assessing social support from humans was conducted solely with NP. All the behavioural tests were carried out in a group paradigm to avoid isolation stress and to reflect natural conditions for planned goat-assisted services (goats in a group). The goats were brought into the experimental arena at least 30 min before testing. If they did not enter voluntarily, they were encouraged by shaking a bucket containing food pellets. After that, they remained alone for another 30 min to avoid the influence of food motivation on the test results.

#### 2.2.2. Assessment of Voluntary Approach to a Human

Testing the voluntary approach to a human consisted of two parts: the passive human test (PH) and the active human test (AH). Based on the modified version of FHAT (Familiar Human Approach Test) proposed for goats [21], the duration of each test was set at 2 min. Both PHs and AHs were conducted 10 times by FP and NP over 10 consecutive days. On each testing day, the goats participated once in the voluntary approach test with FP and once with NP, in a randomised order. The interval between testing with FP and NP was at least one hour. A human zone, which the goats were expected to enter, was marked as a 3 m diameter circle [30] using livestock marker paint at the centre of the experimental arena. The circle was designated three days before the tests to allow the animals to habituate to it. Observations were recorded using the 1–0 sampling method [31] for each individual, noting whether it entered the circle (1) or not (0).

Testing began with the passive human test. When no goat was present inside the circle, the experimenter entered the experimental arena and sat on a low seat at the centre of the circle. It was shown that a human with their head and body oriented towards the goats appears to capture the goats’ attention [32], so each time the experimenters entered the arena, they could sit in a different direction to face the majority of the animals. Once seated, they could not change their position, even if the goats moved to a different location. The experimenters were also instructed to sit upright and refrain from making any distinct movements or speaking to the goats but were allowed to behave naturally (e.g., to scratch themselves if they needed to). When the test ended, the experimenter left the arena for a 5 min break. Once no goat was present in the circle again, the experimenter entered the arena, and the active human test began. The procedure was similar; however, this time the experimenter was allowed to speak to the animals, encouraging them to come closer. Both experimenters had been trained to use a calm voice, similar words, and a comparable pace of speech.

#### 2.2.3. Assessment of Responses to Human Approach

**Figure 2:**
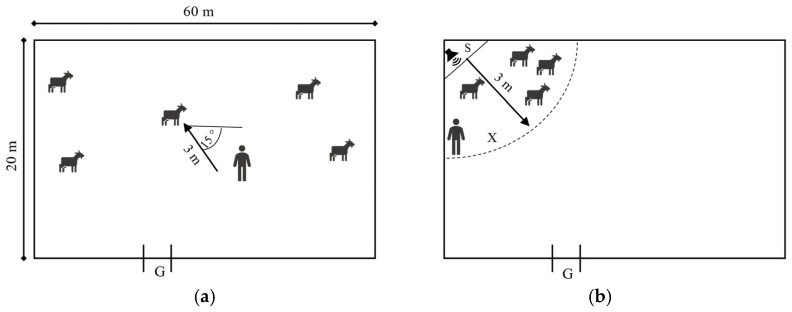
Scheme of the Experimental Zone during (**a**) the Human Approach Test and (**b**) novel sound fear test; (**a**) the experimenters stood at an angle of approximately 15 degrees to a selected goat’s longitudinal body axis, facing it from a distance of approximately 3 metres before starting to approach. Arrow indicate the direction of human approach. The goats could move freely within the Zone; (**b**) the goats stayed in a designated area with provided feed (feeding area) when a sudden sound was played from a remote-controlled loudspeaker (S) located out of the goats’ reach. The human icon indicates the location of the experimenter during the experimental trial. The dashed circles indicate the approximate range of stimulus exposure.

The Human Approach Test (HAT) was conducted following a modification of the Avoidance Distance test described by Mattiello et al. [22] and included assessment of: (1) acceptance of being approached (HAT_A) and then (2) touched by FP and NP (HAT_T). Each test was conducted 10 times by FP and NP over 10 consecutive days. On each testing day, the goats participated once in the approach test with FP and once with NP, in a randomised order. The interval between testing with FP and NP was at least one hour. The experimenters stood at an angle of approximately 15 degrees to a selected goat’s longitudinal body axis, facing it from approximately 3 m (Figure 2a). They then slowly moved forward (about 1 step per second) with their arms resting naturally alongside the torso, the palms of their hands facing downward, directing their gaze toward the animals’ muzzles while avoiding direct eye contact, and attempting to approach, then touch, and stroke the goat for at least 2 sec. During the test, the experimenters were not allowed to speak. Using the 1–0 sampling method [31], it was recorded whether the experimenters approached each goat within 0.5 m without causing it to flee, and whether physical contact was achieved (1—yes, 0—no).

#### 2.2.4. Assessment of Passive Support of a Human During a Novel Sound Fear Test

Since no differences were observed in the goats’ responses to the FP and NF persons in either the HAT or the PHs and AHs (details in the Section 3), it was decided to conduct the novel sound fear test only with one experimenter, given the specifics of the test and the stimuli used (sudden noise as a potential stressor). The choice of the NP was dictated by the fact that, in AAS, goats are more likely to interact with humans with a lower level of familiarity rather than with well-known people.

The last test—the novel sound fear test—was carried out to check whether human presence may mitigate the fear response to sudden sounds, thus whether humans may provide social support for the goats in stressful situations, potentially present during AAS. In one corner of the experimental arena, a quarter-circle with a radius of 5 m was marked with livestock marker paint (Figure 2b). The animals were allowed to enter the experimental arena three days before the tests to habituate to it. In the corner of the quarter circle, a concealed loudspeaker was placed behind a partition in such a way that the distance from the partition to the edge of the circle was three metres. One minute before the test, goat-favoured pellets were scattered within the marked area (feeding area) to ensure that all individuals entered its boundaries. A 10 s recording of an unfamiliar sound at 50 dB was then played, and the goats’ reactions were recorded for two minutes. In the control trial, the goats remained alone in the feeding arena (test F), whereas in the experimental trial, an NP sat on a low seat within the feeding area near the edge of the quarter circle (test H). The person behaved as during the passive human test. To minimise the risk of habituation, two sounds played in a randomised order were used: (1) a noise resembling radio interference with clicks and crackles, and (2) applause. The control and experimental trials were conducted once a day, with at least one hour between trials, in random order, for five consecutive days. For each goat, the time spent in the designated area [s], the occurrence or absence of fleeing after the sound playback (1–0 method [31]), and the latency to return to the feeding arena [s] were recorded. Fleeing or leaving the designated area was defined as having at least two forelimbs outside the feeding area. Return was defined as having at least two forelimbs back inside the feeding area. If a goat did not enter the area within two minutes (120 s), a latency time of 121 s was assigned.

### 2.3. Statistical Analysis

Statistical analysis was performed using SAS 9.4 software (SAS Institute, Cary, NC, USA). The data were not normally distributed, as assessed by the Shapiro–Wilk test.

Binary data were analysed as the probability of an event using the GLIMMIX procedure, applying a generalised linear model with a logit link function. The model included the fixed effects of the experimental factor (feeding vs. neutral human in the interaction tests), sex of the individuals, and the order of experimental repetition (tests conducted on consecutive days). *p*-values for estimated differences were adjusted using Tukey’s correction.

For traits with a continuous distribution, a logarithmic transformation was applied, and the data were analysed using the GLIMMIX procedure. The model included, as above, the fixed effect of the experimental factor (presence vs. absence of a human in the fearfulness test), the fixed effect of sex, and the order of the trial.

Sex was a significant factor only for certain traits: voluntary approach to passive human (PH), acceptance of human approach (HAT_A), and touch (HAT_T). Therefore, in the remaining cases, the effect of sex was not discussed in the results. The order of repetition was not a significant factor. Continuous data were presented as means ± SE, and differences were considered statistically significant at *p* < 0.05.

## 3. Results

### 3.1. Passive Human Test (PH) and Active Human Test (AH)

**Figure 3:**
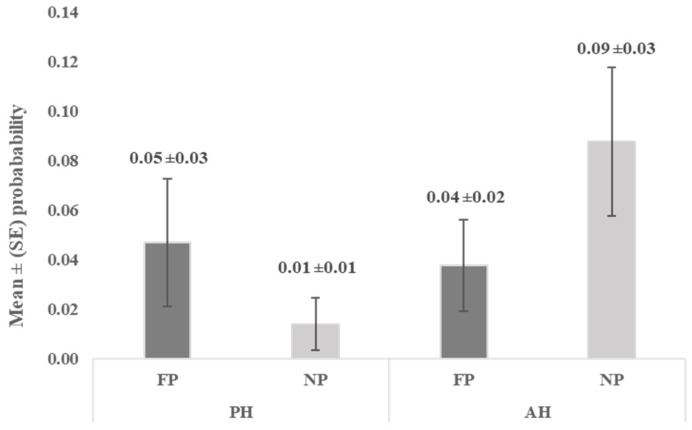
Mean (±SE) probability of approaching the feeding (FP) and the neutral person (NP) in the passive human test (PH) and active human test (AH).

Results obtained from both the passive human test (PH) and active human test (AH) showed no statistically significant differences between responses to feeding (FP) and neutral person (NP) (*p* = 0.0787; *p* = 0.1181, respectively). In each case, the probability of voluntarily approaching either FP or NP was very low (Figure 3).

### 3.2. Human Approach Test (HAT)

**Figure 4:**
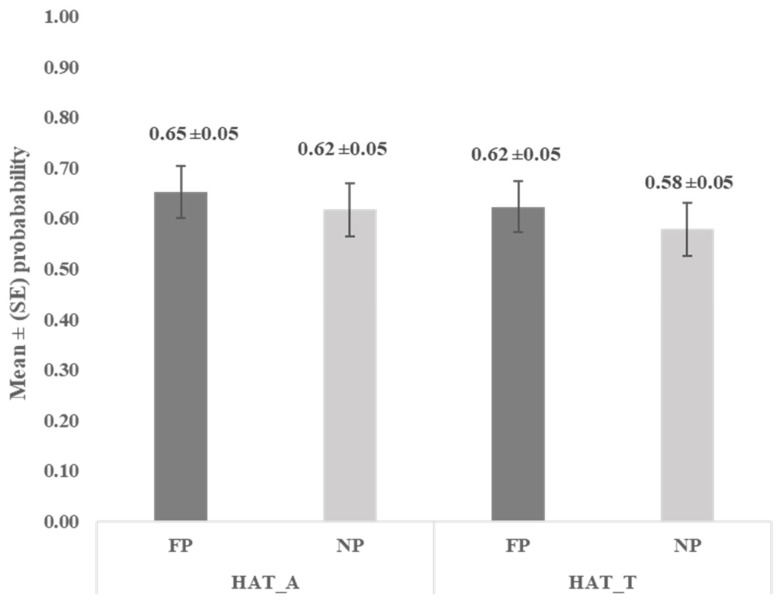
Mean (±SE) probability of accepting human approach (HAT_A) and touch (HAT_T) in the Human Approach Test (HAT); FP—feeding person, NP—neutral person.

Similarly, in the Human Approach Test, no significant differences were noted between FP and NP in acceptance of human approach and touch (*p* = 0.6377 and *p* = 0.5437, respectively). Both the feeding and neutral person approached and touched the goats with a relatively high probability (Figure 4).

### 3.3. Novel Sound Fear Test

**Figure 5:**
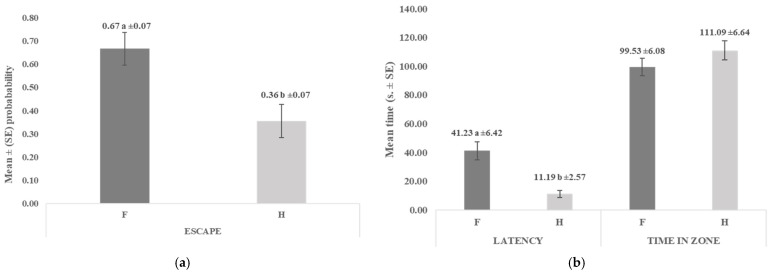
Responses of goats in the novel sound fear test when the human was absent (F) or present (H): (**a**) mean (±SE) probability of escaping after sound playback; (**b**) mean latency time (s. ± SE) to return to the feeding zone after sound playback; mean time in zone (s. ± SE)—total time each goat spent in the feeding zone from the moment of sound playback (max. 2 min). Means marked with different letters differ significantly at *p* < 0.01.

The analysis of escape behaviour in the novel sound test revealed a significant difference between the control (F; only feed, human absent) and experimental conditions (H; human present) (*p* = 0.0047). In the control trial, escape responses occurred with a significantly higher probability than in the condition with the human (Figure 5a). Latency times to return to the feeding zone after sound playback also differed significantly between the two conditions (*p* < 0.0001). Goats returned faster in the presence of humans than in their absence. However, no significant difference was found in the time spent in the feeding zone (*p* = 0.3201) (Figure 5b).

### 3.4. Sex Differences

Significant differences between male and female goats were found for PH, HAT_A, and HAT_T (Table 1). Females voluntarily approached a passive human (PH) with a higher probability than males (*p* = 0.0164) and were more likely than males to accept being approached (HAT_A; *p* < 0.0001) and touched (HAT_T; *p* < 0.0001).

## 4. Discussion

Voluntary engagement in interactions with humans is a key characteristic required of animals participating in AAS, as the animal’s motivation and enjoyment of the task make the experience particularly pleasant for all parties involved [33]. It also has important implications for animals’ welfare [7], especially given that animals are incorporated into many different types of activities with patients [4]. Therefore, the history of socialisation with humans is important for understanding the involvement of animals in AAS [15,17,18,19]. It was assessed whether goats that did not undergo early intensive socialisation with humans, such as petting, training, or playing, and received standard husbandry care would show predispositions to AAS in terms of sociability towards humans based on simple human-interaction tests. Additionally, taking into account interactions with different people during incorporation into AAS, it was examined whether, at the initial stage of training, test outcomes might depend on the person conducting the tests. Due to the relatively low level of socialisation with humans in the studied goats, responses to a caretaker (daily contact, possible association with feeding) and to a neutral person (familiar but less frequent contact, no feeding context), rather than to a completely unfamiliar person, were compared.

The goats accepted a human approach with a probability of 0.65 for a feeding person and 0.62 for a non-feeding (neutral) person. A similar trend was observed for acceptance of touch, which was 0.62 and 0.58, respectively. Hence, in the majority of cases, the goats accepted humans in their individual space and did not perceive them as a threat, despite the lack of early, intensive socialisation with humans. In the study conducted by Mattiello et al. [22], the experimenter could approach and touch 45.8% of the goats. The authors also observed that avoidance distances were shorter, whereas the frequency of contact with humans was higher on small than on large farms. Since none of the individuals in the current study were ever kept on a large farm, their contact with humans might have been more frequent. In the context of further training for AAS, the goats from the current study were to be kept in a small group of ten individuals and would therefore have good opportunities to interact with people. As noted by different authors [13,34,35], not only the frequency, but also the manner in which animals are handled shapes the human–animal relationship on farms and may be reflected in the animals’ behavioural responses to humans during specific tests. At this stage of the study, the goats were subjected only to standard handling procedures; intensive human socialisation and training related to their future participation in AAS had not yet been introduced, and any additional interactions with humans were solely voluntary. Nonetheless, even under such conditions, humans can already influence the formation of relationships with animals, which strongly affect animal welfare [14,36]. Given the decision to incorporate goats into AAS, the results of the Human Approach Test are not fully satisfactory. Avoidance responses to approaching humans in some trials might have indicated not only unwillingness to interact with them, or lack of motivation [33], but also discomfort and stress in situations of forced human presence [37]. It is the aspect that would have to be considered in future training. Ultimately, individuals who continue to avoid contact with humans should be excluded from AAS preparations due to welfare concerns [7]. It is also an important issue from the client’s perspective. For example, when animals walk away and do not wish to engage, young adolescents with low self-esteem may perceive this as a rejection by the animal [33]. Since clients cannot be supported at the expense of animal welfare, only individuals with an appropriate disposition and proper training should be selected for AAS [2,7].

Unlike in the Human Approach Test, the goats in the present study rarely approached a human voluntarily in both the passive human test (PH) and active human test (AH). For a feeding person, the probability of successful trials was 0.05 and 0.04, respectively, and for a neutral person, 0.01 and 0.09, respectively. Hence, although the lack of intensive human socialisation alongside standard handling procedures generally mitigates fear in the presence of humans, as shown in the Human Approach Test, it remains insufficient to promote the animals’ willingness and motivation to engage in voluntary interactions with humans. Early life exposure to humans results in goats that are gentler, calmer, and more comfortable in the presence of people than goats with little or no human contact [17]. An early study comparing hand-reared and dam-reared kids showed that the latter exhibited greater avoidance distances from humans and were more fearful [38]. Later studies confirmed that human rearing of goat kids results in better human–animal relationships than dam rearing [39], greater confidence in kids toward humans, and greater ease of management in adulthood [40]. However, adult goats can also improve their sociability towards humans through frequent, positive treatment [40,41,42]. Regarding the goats in the current study, various forms of tactile interaction may be useful for further training in preparation for the AAS. Massaging was shown to promote goats’ relaxation and improve the quality of human–animal interactions [43], brushing the goats had a positive effect on their emotional state [44], and stroking the goats was shown to be effective for achieving a positive handling treatment [42]. Positive human emotional facial expressions also stimulated approach and interaction in goats [45]. Similarly, inviting goats to approach humans can be achieved by offering food [46] or by making slow arm and hand movements [47]. To attract goats’ attention and maintain positive handling, talking softly to the animal and using vocal calls such as ‘come on’ were also used [46,47,48]. However, significant differences in goats’ approaching a human between passive and active human tests were not observed when the experimenter motivated the goats with a soft voice. It is likely that the relatively short habituation period (one month) and the limited human–goat contact were insufficient to elicit vocalisations that would attract the goats. Langbein et al. [12] did not observe that short-term positive handling affected goats’ human-directed behaviour (e.g., gaze or contact alternations with the experimenter) during the unsolvable task. The goats did not differ in their responses from those in the standard husbandry group. On the contrary, the authors suggest routine husbandry practices alone may be enough for farm animals to perceive humans as points of reference in difficult situations.

Several differences were found between male and female goats in the level of their sociality toward humans. Females voluntarily approached a passive human (PH) with a higher probability than males and were more likely than males to accept being approached and touched by a human. Previous studies have also reported sex-related differences in sociability and willingness to interact with humans in favour of females, for example, in dogs [49,50,51] and pigs [52]. However, other studies did not report any sex differences in this context, for instance, in calves [53] and between heifers and young steers [54]. The different responses of male and female goats observed in the current study may also result from potentially different experiences with humans, which were not fully known to the authors. Additionally, these differences may be related to individual personality traits, which could have influenced the goats’ responses to humans [55].

In the novel sound fear test, the goats escaped significantly less often after sound playback and definitely needed less time to return to the feeding zone when a human was present. These results suggest that humans may have served as social support for goats in stressful situations. Social buffering was observed in farm animals in previous studies. Human presence was shown to diminish stress response in horses during habituation to novel objects [56] and to reduce stress reactions in isolated cows [18]. In turn, Scandurra et al. [19], who examined human social buffering in goats and dogs with limited prior socialisation with humans, noted that both species could benefit from social buffering if human interactions had been accepted. The goats in the current study mostly accepted a human approach but were unwilling to initiate interaction with a human partner. However, the results of the fear test indicate that these short-term interactions were sufficient for the human’s presence to provide support during a stressful event. This finding is promising, given the potential participation of the studied individuals in AAS, which may involve some level of stress [9]. It is important that animals feel safe with their caretaker during AAS sessions [10]. In the current study, the time spent in the feeding zone did not differ between the tests with and without a human present, which may be related to the seat’s placement. It was positioned near the edge of the feeding zone, meaning that even if the goats left the feeding area, they could still remain close to the human. This factor should be taken into account in future studies.

It is expected that the goats would accept being approached and touched by a feeding person and would also approach him more willingly than a neutral person, due to more frequent interactions during daily routines and the possible association of the person with feeding. Farm ungulates can associate people with pleasant events such as feeding, stroking, or grooming, and may discriminate between humans [57,58]. For example, it was shown that lambs tended to interact more with their stockperson than with a familiar person who spent an equal amount of time near their enclosure, dressed in similar clothing [59]. However, no differences in goats’ responses to the two humans were observed across any condition, contrary to predictions. As demonstrated by Tallet et al. [60], gentle, positive interactions such as stroking promoted the development of lambs’ affinity for their caretaker and could provide calming and rewarding experiences for the animals without the use of food. Nonetheless, previous stroking, combined with feeding from a milk bucket, led to increased time spent near the bucket when the caretaker entered the home pen during a test. Hence, it might have been connected to associating a human with food. Unlike during the habituation period in the current study, when the goats willingly approached a caretaker holding a bucket of feed, they were not motivated to approach either person during testing (no visible food). This general lack of interest may stem from limited socialisation with humans earlier in life, while the absence of differences between a feeding and neutral person could be due to the short period (one month) of standard handling by the current caretaker, which may not have been sufficient to form a bond or develop an affinity for a particular human. Therefore, it cannot be definitively determined whether the goats did not prefer his presence or did not associate him with feeding.

In addition to the possible explanations for the goats’ low interest in interacting with humans discussed above, it is worth noting that the tests were conducted in the external arena, which normally served as a small pasture for the animals. While on pasture, goats are generally farther from humans, which may reduce test sensitivity [21]. Therefore, the goats in the current study might not have been motivated to interact with humans, as they had access to grass and remained in a group with their conspecifics. This factor must be included when evaluating the animals for AAS. It is possible that certain elements of the pasture or contexts of human behaviour were associated by the goats with their previous housing environments, which may also have influenced the test results. The major limitation of the current study is actually a lack of a control trial-goats highly socialised with humans, which would support the discussion on the results. However, this particular study was part of a training protocol for goats that arrived at the research facility from different environments and had only standard human contact, which reflects the real-world conditions under which animals are often considered for AAS. While such conditions can provide valuable insights for AAS practitioners and those involved in preparing animals for AAS, they also make replication of the study more challenging. Further research is needed to determine whether introducing intensive and positive interactions with humans would make a difference. At this point, only potential predispositions can be discussed. Other limitations that must be considered when analysing the current study include the small number of individuals tested and the limited knowledge of their life history and prior experiences with humans.

## 5. Conclusions

The analysed group of goats without prior intensive socialisation with humans and kept under standard handling conditions showed some predispositions to participate in animal-assisted services (AAS) in the context of human–animal interactions. The goats showed signs of perceiving humans as a source of support during the novel sound fear test and mostly accepted being approached and touched both by the caretaker and a neutral person. These conditions are important to ensure the welfare of both the animals and the human clients during AAS sessions. However, a lack of interest in voluntary interactions with humans, which is crucial in AAS, constitutes a factor that excludes animals from engagement in this area. Based on available literature, further training for the goats in this study should include frequent, positive interactions with humans to improve their acceptance of being approached and their willingness to voluntarily initiate contact with people. The lack of differences in responses to the caretaker and to a neutral person may result from limited prior socialisation with humans, previous experiences in human interactions, or the short habituation period in the present study, which may have been insufficient to elicit differentiated responses of the goats toward different people.

## Figures and Tables

**Table 1:** Responses of male and female goats in interaction tests. PH—passive human test; HAT_A—Human Approach Test—acceptance of being approached; HAT_T—Human Approach Test—acceptance of touch; mean—mean probability of successfully completing the test; SE—standard error; *p*—probability value.

Test	Sex	Mean	SE	*p*
PH	Male	0.01	0.01	0.0164
	Female	0.09	0.03	
HAT_A	Male	0.43	0.05	<0.0001
	Female	0.80	0.04	
HAT_T	Male	0.43	0.05	<0.0001
	Female	0.75	0.04	

## Data Availability

The original contributions presented in this study are included in the article. Further inquiries can be directed to the corresponding author.

## Abbreviations

The following abbreviations are used in this manuscript:

AAS	Animal-Assisted Services
IAHAIO	International Association of Human–Animal Interaction Organisations
HAI	Human–Animal Interaction
HAT	Human Approach Test
AWIN	The European Animal Welfare Indicators Project
FP	Feeding person, main caretaker of the animals
NP	Non-feeding, academic staff, one of the visitors to the Animal Zone
PH	Passive human test
AH	Active human test
FHAT	Familiar Human Approach Test
HAT_A	Acceptance of being approached
HAT_T	Acceptance of being touched
